# The ErbB-tyrosine kinase inhibitor, neratinib, has anti-inflammatory effects in multimorbidity by increasing macrophage efferocytosis via the upregulation of MerTK

**DOI:** 10.1186/s12950-026-00495-9

**Published:** 2026-03-19

**Authors:** Kieran A. Bowden, Carl Wright, Susan Clark, Tomas Fernandez Correa, Simon A. Johnston, Helen M. Marriott, Sheila E. Francis, Lynne R. Prince

**Affiliations:** School of Medicine and Population Health, University of Sheffield, Beech Hill Road, Sheffield, S10 2RX UK

**Keywords:** Neutrophils, Macrophages, Inflammation, Lung disease, Multimorbidity, Efferocytosis, Apoptosis, ErbB, MerTK

## Abstract

**Background:**

Chronic inflammatory disease is responsible for huge and increasing global mortality and morbidity. Unregulated inflammatory cells, including neutrophils and macrophages, are major drivers of chronic inflammatory disease. Efferocytosis plays a critical role in inflammation resolution by removing effete inflammatory cells from tissues. Despite defective efferocytosis being critical in inflammatory disease progression there are no therapies to correct these defects in clinical use. Here, using experimental models of atherosclerosis and lung injury, we identify the ErbB family tyrosine kinase inhibitor (TKI), neratinib, as a putative efferocytosis-targeting therapy.

**Results:**

In an experimental model of atherosclerosis and lung injury, two doses of neratinib significantly increased efferocytosis in the lungs of mice concomitant with a reduction in the proportion of lung neutrophils. Neratinib significantly increased human neutrophil apoptosis and efferocytosis of apoptotic neutrophils by monocyte-derived macrophages (MDMs). In addition to increased efferocytosis, neratinib treated macrophages demonstrated both increased phagocytosis and macropinocytosis. Neratinib increased MDM surface expression of the efferocytosis receptor MerTK independent of protein synthesis and transcription which correlated with elevated efferocytosis in MDMs and inhibitors of MerTK blocked neratinib-induced efferocytosis.

**Conclusions:**

Thus, we describe a novel role for neratinib in driving efferocytosis in multimorbidity and suggest that ErbB TKIs may have therapeutic potential in inflammatory disease by restoring macrophage function and promoting inflammation resolution.

## Background

Macrophages are the most abundant immune cells in the lung and play a vital role in regulating homeostasis, infection and inflammation via the clearance of pathogens and effete cells including inflammatory neutrophils [1, 2]. The ingestion of apoptotic cells by macrophages, termed “efferocytosis,” not only removes potentially damaging cells from the lung, but also triggers an anti-inflammatory phenotype in the macrophage, further driving inflammation resolution [3]. It is widely known that efferocytosis is impaired during ageing, by inflammatory factors and in inflammatory diseases including chronic obstructive pulmonary disease (COPD), asthma and cardiovascular disease (CVD), which increases the burden of uningested cells including neutrophils [4–7]. COPD and CVD are common components of multimorbidity syndromes, defined as the presence of two or more chronic diseases, which have become increasingly prevalent over the last two decades [8].

Apoptotic neutrophils not removed from sites of inflammation via efferocytosis can lead to secondary necrosis, resulting in the release of pro-inflammatory cytokines, as well as cytotoxic granular components and reactive oxygen intermediates. This process leads to tissue damage and perpetuation of the inflammatory response. Understanding how these pathophysiological mechanisms can be manipulated and identifying a class of compounds to therapeutically modify these pathways would be of significant benefit, particularly in the case of chronic inflammatory diseases where ineffective clearance of apoptotic neutrophils is accepted to be a contributing factor in driving disease [9–11].

The ErbB tyrosine kinases, consisting of ErbB1/epidermal growth factor receptor (EGFR), ErbB2/HER2, ErbB3, and ErbB4, are cell surface growth factor receptors that promote cell growth and survival [12]. We have previously shown that targeting ErbBs genetically and pharmacologically has anti-inflammatory effects in models of neutrophilic inflammation including by reducing neutrophils at the site of inflammation in zebrafish models and increasing macrophage efferocytosis in the murine lung [13, 14]. Our phosphoproteomic analysis identified an enrichment in the Rho GTPase cycle, a known controller of efferocytosis and phagocytosis [14]. In support of this, our work in *Drosophila* to overexpress the EGFR ligand, Spitz, reduces efferocytosis in fly embryo hemocytes, implicating ErbBs as key regulators in these cellular pathways [15]. ErbBs are overexpressed or mutated in many cancers, especially breast, ovarian and non-small cell lung cancer, and are a key target in the development of cancer therapies [16, 17]. Neratinib is one of a number of ErbB-TKIs that are approved for the treatment of cancer and could be potentially be reprofiled for inflammatory diseases [18]. Here we study the effects of neratinib in human neutrophils, MDMs and a murine model of multimorbidity to understand the direct effects of neratinib on phagocytosis and the mechanisms by which this may occur.

## Methods

### Primary cell isolation from human blood

All in vitro experiments using primary human cells isolated from healthy participants were performed in compliance with the University of Sheffield Research Ethics Committee (study number: 031773). Neutrophils and peripheral blood mononuclear cells (PBMCs) were isolated by dextran sedimentation and plasma/Percoll™ phase separation centrifugation as previously described [19]. Briefly, blood (+ 3.8% tri-sodium citrate (1:10 v/v)) was centrifuged at 270 g for 20 min (all centrifugation was performed at room temperature); separating the blood into platelet-rich plasma (PRP) and cells. The PRP was transferred to a 50 ml tube and centrifuged at 750 g for 20 min, yielding platelet-poor plasma (PPP). To the blood, 6 ml of 6% dextran (w/v in saline) (Merck; D1037) was added before being left at room temperature for 30 min to allow the red blood cells to sediment. The upper layer, containing white blood cells (WBCs), was gently aspirated and centrifuged at 200 g for 6 min. The WBC pellet was gently resuspended in 2 ml of PPP and overlaid onto a plasma/Percoll™ gradient (the lower phase comprising of 1.02 ml of 90% Percoll™ (v/v in saline) (GE Healthcare; 17-0891-02) and 0.98 ml PPP and the upper phase comprising of 0.84 ml 90% Percoll™ and 1.16 ml PPP). The gradient was centrifuged at 230 g for 11 min without breaking. Centrifugation yielded three distinct layers of: PBMCs, granulocytes (typically comprised of > 97% neutrophils) and red blood cells. PBMCs were washed and resuspended in RPMI 1640 media (Lonza; 12–702 F) + 1% Penicillin-Streptomycin at a concentration of 5 million cells/ml. Neutrophils were washed and resuspended in media (RPMI 1640 + 10% foetal calf serum (FCS) (ThermoFisher; A3840001) + 1% Penicillin-Streptomycin) at a density of 5 million cells/ml.

### Assessment of cell differentials and neutrophil apoptosis

To assess the morphology and condition of isolated cells, staining and analysis of cells was performed by transferring and fixing cells onto glass microscope slides by cytocentrifugation. Cell populations were gently pipetted in order to resuspend cells evenly throughout the solution and transferred to an assembled cytocentrifuge funnel, filter paper and microscope slide. Samples were cytocentrifuged at 300 rpm for 3 min. Cells were fixed with methanol and stained in Kwik-Diff Solutions (Thermo scientific; 9990706–9990707) before addition of a coverslip and imaging of cellular morphology by light microscopy.

### Differentiation of MDMs

PBMCs isolated as above were seeded onto sterile glass coverslips in a 24-well plate containing at 2.5 million PBMCs per well and incubated at 37 °C/5% CO_2_ for 1 h to allow monocytes to adhere to the coverslips. Following this, contaminating cells were removed using a Pasteur pipette. Media was replaced with RPMI 1640 containing 10% FCS, and 1% penicillin-streptomycin was added to wells and cells incubated at 37 °C/5% CO_2_. Media was replaced every 2 days for 7–10 days to allow differentiation into MDMs [20].

### Efferocytosis and bead phagocytosis assays

MDMs were differentiated as above and incubated with reagents as detailed in the results section. For efferocytosis assays, neutrophils were cultured for 22–24 h to induce apoptosis (mean apoptosis: 91.76%) and stained using the phosphatidylserine probe, annexin V PE (BioLegend; 640908) as per manufacturer’s instructions. Two and a half million neutrophils were added to each well containing MDMs (approximately ten neutrophils per MDM) and incubated at 37 °C/5% CO_2_ for 1 h to facilitate efferocytosis. Following this, media was removed and each well was rinsed five times with ice cold phosphate-buffered saline (PBS) to remove all uningested neutrophils. MDMs were then fixed with 4% paraformaldehyde and stained with 100 µM FITC-Phalloidin (AAT Bioquest; 23115). Coverslips were removed from the wells and were transferred to labelled, glass microscope slides containing a drop of VectaShield. Efferocytosis rates were determined by quantifying the number of MDMs containing apoptotic neutrophils by taking images using a ZOE fluorescent imager (Bio-Rad) using a x20 objective lens and analysing the images with imageJ software. MDMs were determined to be containing apoptotic neutrophils (and therefore performing efferocytosis) if any red apoptotic neutrophils were co-localised with the green signal produced by FITC-phalloidin stained MDMs. Counts were performed manually within Image J. The threshold function within Image J was utilised to make annexin V signals clearer to perform manual counting.

For phagocytosis assays, 2 μm fluorescent latex beads (Merck; L3030) were resuspended in media at a density of 5 million beads/ml, and 2.5 million beads were added to each well of MDMs and incubated for 1 h at 37 °C/5% CO_2_ to permit phagocytosis. Following this, media was removed and each well rinsed five times with ice cold PBS to remove all uningested beads. MDMs were fixed with 4% paraformaldehyde, stained with FITC-Phalloidin, and mounted onto microscope slides as above. Phagocytosis rates were determined by quantifying the number of MDMs containing fluorescent beads by analysing images obtained using a ZOE fluorescent imager, followed by manual analysis in the software ImageJ.

### MDM macropinocytosis assay using fluorescent dextran and flow cytometry

MDMs were differentiated as described above and incubated with media (control) or neratinib (20 µM) (Cayman Chemical Company; 18404) for 20 h. Following this, fluorescently labelled dextran (100 ng/ml; 70 kDa) (Invitrogen: D1822) was added to cells and incubated at 37 °C/5% CO_2_ for 1 h. MDMs were removed with ice cold PBS and centrifuged at 575 g for 6 min. The pellet was resuspended in 1 ml PBS and 20 nM Thiazole Red (TOPRO3, a viability stain) was added to relevant tubes immediately before flow cytometry. Cells were analysed by a Cytek Aurora 3 laser flow cytometer and CYTEK SpectroFlo software. Cells were first determined to be viable based on TORPRO3 negativity and subsequently analysed for fluorescent dextran derived signals.

### Quantifying MerTK (Mer receptor tyrosine kinase) cell surface expression on MDMs by flow cytometry

MDMs were differentiated in 24 well plates as described above and treated with reagents as detailed in the results section. Cells were removed from wells by placing 24-well plates on ice and washing wells vigorously five times with ice cold PBS. MDMs were transferred to tubes containing blocking buffer (5% bovine serum albumin (BSA), 5% rabbit serum in PBS) and incubated for 1 h (37 °C/5% CO_2_) before a further incubation with an anti-MerTK antibody (diluted 1:50) (Abcam; ab307522) at 4 °C for 30 min in the dark, following which the viability dye propidium iodide (PI) was added to relevant tubes to achieve a final concentration of 10 µg/ml. Unstained and single-stained controls were also included. Cells were analysed by a Cytek Aurora 3 laser flow cytometer and Cytek SpectroFlo software. Cells were first determined to be viable on the basis of PI exclusion and subsequently analysed for MerTK expression.

### Measuring MerTK expression by live imaging

To observe the expression of MerTK in healthy human MDMs following treatment with neratinib, MDMs were differentiated as described above and incubated with or without neratinib for 2, or 20 h. Media was removed and 1 ml of blocking buffer (5% BSA, 5% rabbit serum, 85% PBS) was added and cells were incubated for 1 h at 37 °C/5% CO_2_. Each well was then washed with PBS three times and 200 µl of 1:250 MerTK antibody diluted in antibody diluent (1% BSA in PBS) was added to each well, before being incubated at 4 °C for 30 min in the dark. Following MerTK staining, cells were visualised using a ZOE fluorescent imager.Images were analysed using ImageJ software, utilising the threshold function to identify areas positive for fluorescent signals, followed by use of the analyse particle function to quantify the area of each cell which was positive for MerTK signal. Cell numbers were validated by manual counts.

### Murine in vivo model of multimorbidity

#### Mouse husbandry

All in vivo work was approved by the Sheffield ethical review board and the UK Home Office PPL: PP0517861 and all handling and procedures were performed by fully trained staff. All present studies were performed using male C57BL/6J mice.

### Murine atherosclerosis and acute lung injury multimorbidity model

The recombinant adeno-associated virus (rAAV) AAV-mPCSK9 (Addgene; Jacob Bentzon: Plasmid #58376) and a Western diet was used to induce atherosclerosis in mice. Mice were injected with 100 µl (3 × 1010 vg) of AAV-mPCSK9, which encodes for gain-of-function mutant form of the PCSK9 gene (reducing low-density lipoprotein clearance), using a 1 ml sterile pipette via a single tail vein injection. This was performed on day 1 of the investigation. On day 8 of the investigation, all injected mice were introduced to a Western diet (WD 88137) for the remainder of the investigation for the development of atherosclerosis. On day 96 of the investigation, mice were anesthetised and exposed to 7 µg lipopolysaccharide (LPS) from *Escherichia coli* O26:B6 (Merck; L8274) (dissolved in in 50 µl PBS) intranasally to develop acute lung injury. This was performed dropwise by slowly transferring the solution to the nose via a sterile pipette. On days 97 and 98 of the investigation, mice were dosed with PBS (control group) or 20 mg/kg neratinib, dissolved in methylcellulose (0.5% w/v 400 cp. methylcellulose + 0.4% Tween-80 (polysorbate) in sterile water) via oral gavage.

### Extraction and processing of bronchoalveolar lavage fluid (BALF) samples from mice

On day 85 of the investigation, all mice were sacrificed and bronchoalveolar lavage performed by inserting a catheter disinfected with 70% ethanol into the trachea of the sacrificed mouse by surgically opening the neck of the mouse and aligning the trachea using cotton thread. A 1 ml syringe was used to pump 100 µl of PBS into the thorax of the mouse. The lavage fluid was collected in the syringe and transported to a 15 ml falcon tube on ice. This process was repeated 3 times on each mouse to extract all lavage fluid. BALF was centrifuged and cells cytocentrifuged onto cytocentrifuge slides as described above.

### Differentiating murine bone marrow-derived macrophages (BMDMs)

Bone marrow from mouse femurs were differentiated into BMDMs in the presence of L929 conditioned media. Femurs were removed and placed in sterile PBS. A 0.5 ml microcentrifuge tube was placed inside of a 2 ml microcentrifuge tube, and a hole was made in the bottom of the 0.5 ml microcentrifuge tube using a 25-gauge needle. To both microcentrifuge tubes, 200 µl of sterile PBS was added. Using surgical scissors, the caps of each end of the femurs were cut and the bones were placed into the 0.5 ml microcentrifuge tubes, which were subsequently placed inside 2 ml microcentrifuge tubes. All tubes were centrifuged at top speed for 30 s in order to flush the bone marrow out of the femur, and into the 200 ml PBS in the bottom of the 2 ml microcentrifuge tubes. The PBS was removed, and the pellets were resuspended in RPMI 1640 (+ 10% FBS, + 10% L929 fibroblast supernatant) at a density of 2.5 × 10^5^ cells/ml. The cells were incubated at 37 °C/5% CO_2_ for 5 days. Following this, non-adherent cells were removed, and fresh media was added and then changed twice per week.

### Statistical analysis

All data were analysed using GraphPad Prism and Microsoft Excel. Statistically significant differences are indicated in figurelegends. Where data represented *n* ≥ 3 statistical analysis was performed on all datasets. A Shapiro-Wilks normality test was conducted on all appropriate datasets to determine whether a parametric, or non-parametric, statistical test should be used. In experiments where only one variable was being investigated, a one-way ANOVA was used. When directly comparing the means of two groups, a student’s t-test was utilised. Post analysis, multiple comparisons tests were commonly employed to further identify variations between groups being tested. Notably, Dunnett’s post-test multiple comparison tests were undertaken for these purposes.

## Results

### Neratinib treatment reduces the proportion of neutrophils and elevates efferocytosis in the lungs of multimorbid mice

**Fig. 1:**
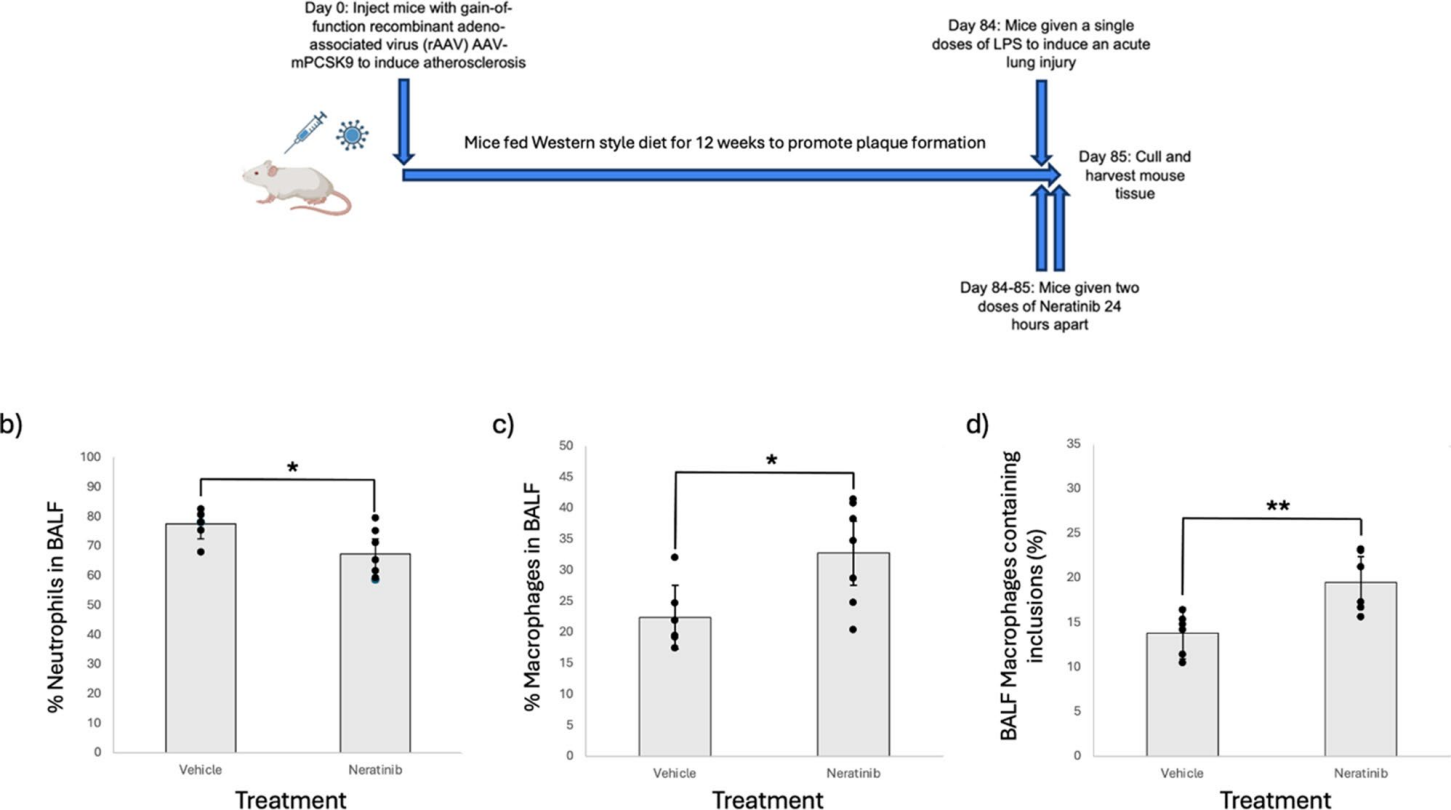
Neratinib has anti-inflammatory effects in a multimorbidity mouse model. Mice injected with PCSK9-AAV8 were fed a Western diet for 12 weeks following which they received intranasal LPS and either treated with neratinib (*n* = 9) or vehicle control (*n* = 9) (**a**). BALF was obtained and the percentage of neutrophils (**b**) the percentage of monocytes (**c**) and macrophage efferocytosis (**d**) were quantified by light microscopy. Bars indicate mean, error bars indicate standard deviation. Each icon represents one mouse. Statistical analysis was performed by using an unpaired Students’ t-test, p values are as indicated; *: *p* < 0.05, **: *p* < 0.01

Neutrophils drive a number of inflammatory processes, many of which coexist in multimorbidity. For example, people with COPD are twice as likely to have CVD, both of which are associated with a neutrophil pathology. We have previously shown that ErbB-TKIs reduce neutrophilic inflammation following acute lung injury [13, 14]. To understand, as a proof of concept, whether neratinib could modify neutrophil clearance in multimorbidity we used a mouse model of combined atherosclerosis and LPS-induced acute lung inflammation. Mice injected with PCSK9-AAV8 were fed a high fat diet for 12 weeks following which they received intranasal LPS to induce lung neutrophilia (Fig. 1a). BALF analysed from mice treated with neratinib (20 mg/kg) had a significantly lower proportion of neutrophils in the lungs and airways (Fig. 1b), an increase in the proportion of macrophages (Fig. 1c) and significantly increased macrophage efferocytosis (Fig. 1d), compared to vehicle control mice. These effects are seen after only 2 doses of neratinib given concurrently with LPS.

**Fig. 2:**
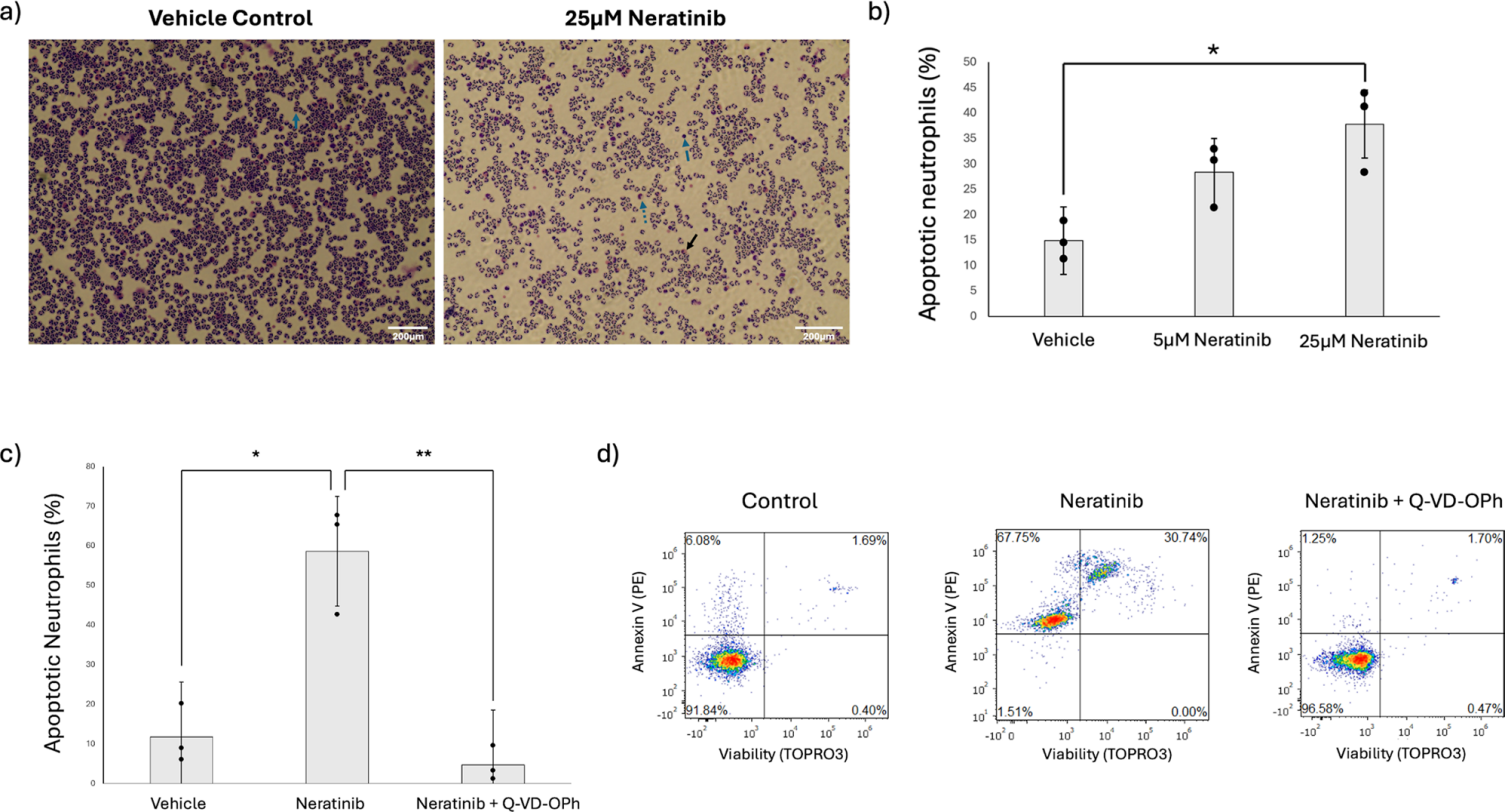
Neratinib significantly induces apoptosis of human neutrophils in vitro in a caspase-dependent manner. Neutrophils isolated from healthy volunteers were incubated for 6 h with DMSO 0.1% (v/v) (vehicle control) or neratinib [5 µM or 25 µM] (**a**-**b**), or with DMSO 0.1% (v/v) (vehicle control), neratinib (25 µM), or neratinib alongside the pan-caspase inhibitor Q-VD-OPh [10 nM] at 37 °C/5% CO_2_ (**c**-**d**). Apoptosis was assessed by light microscopy (**a**-**b**) or flow cytometry (**c**-**d**). Representative light microscopy images showing examples of cytocentrifuge slides (**a**), solid blue arrows highlight viable neutrophils, dashed blue arrows highlight apoptotic neutrophils, dotted blue arrows highlight monocytes, solid black arrows highlight eosinophils. Representative flow cytometry dot plots showing annexin-V/TOPRO3 staining profiles (**d**). Viable cells were dual negative for annexin V and TOPRO3 (lower left quadrant), apoptotic cells were positive for annexin V but not TOPRO3 (upper left quadrant), and secondary necrotic cells were dual positive for annexin V and TOPRO3 (upper right quadrant) (**d**). Data was obtained across three experimental repeats (*n* = 3). Graphs indicate mean ± standard deviation. Statistical analysis was performed by using an ordinary one-way ANOVA with Dunnett’s multiple comparisons, p values are as indicated. *: *p* < 0.05, **: *p* < 0.01

**Fig. 3:**
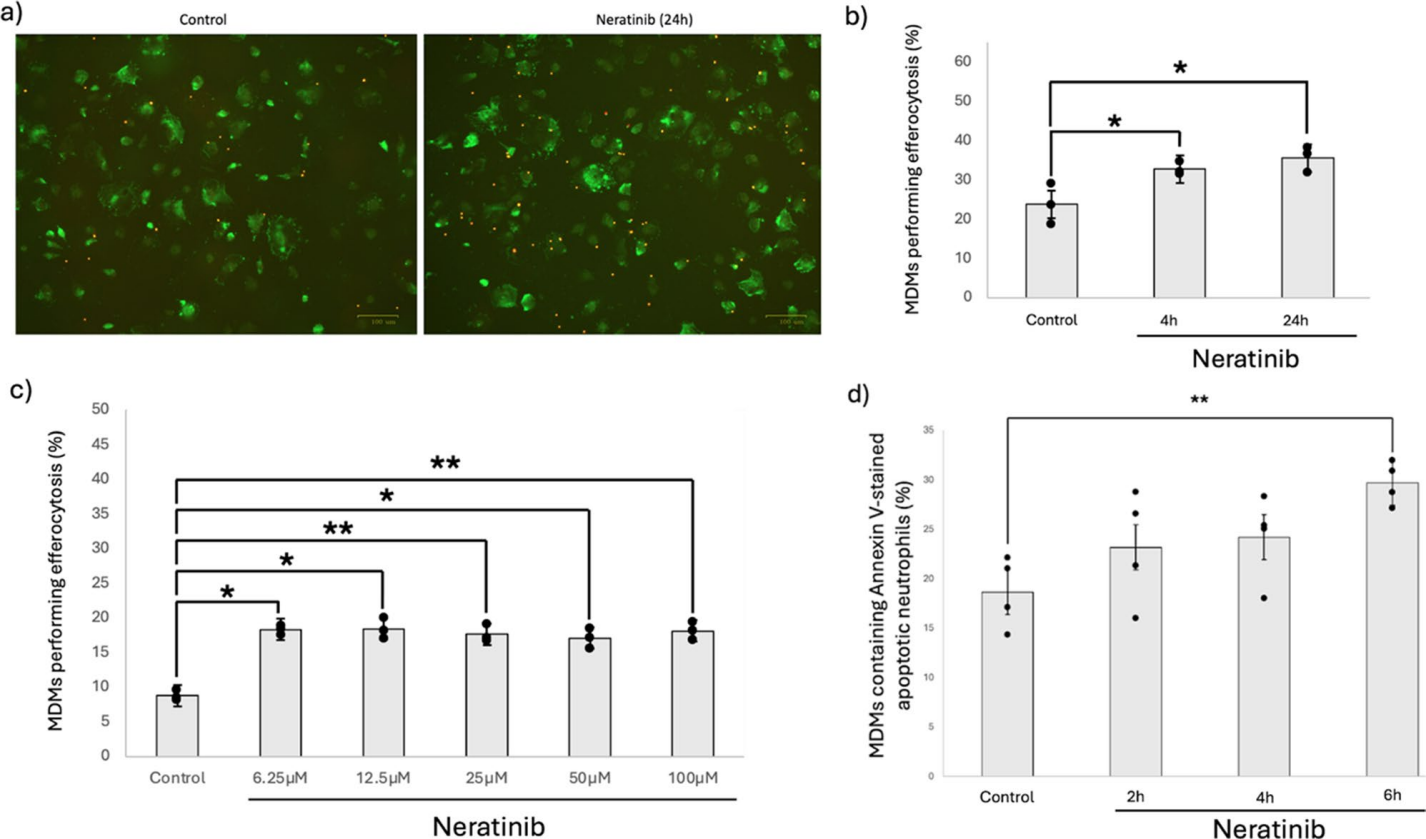
Neratinib increases efferocytosis of apoptotic human neutrophils by MDMs. MDMs were treated with neratinib at the concentrations and times indicated following which they were incubated with annexin-V (PE, red) stained apoptotic neutrophils at 37 °C/5% CO_2_. MDMs were stained with FITC-Phalloidin (green) and efferocytosis quantified by fluorescence microscopy (**a**-**c**, *n* = 3) or flow cytometry (**d**, *n* = 4). Representative fluorescent microscopy images (**a**). Scale bars = 100 μm. Graphs indicate mean ± standard deviation. Statistical analysis was performed by using a one-way ANOVA with Dunnett’s multiple comparisons, p values are as indicated. *: *p* < 0.05, **: *p* < 0.01

**Fig. 4:**
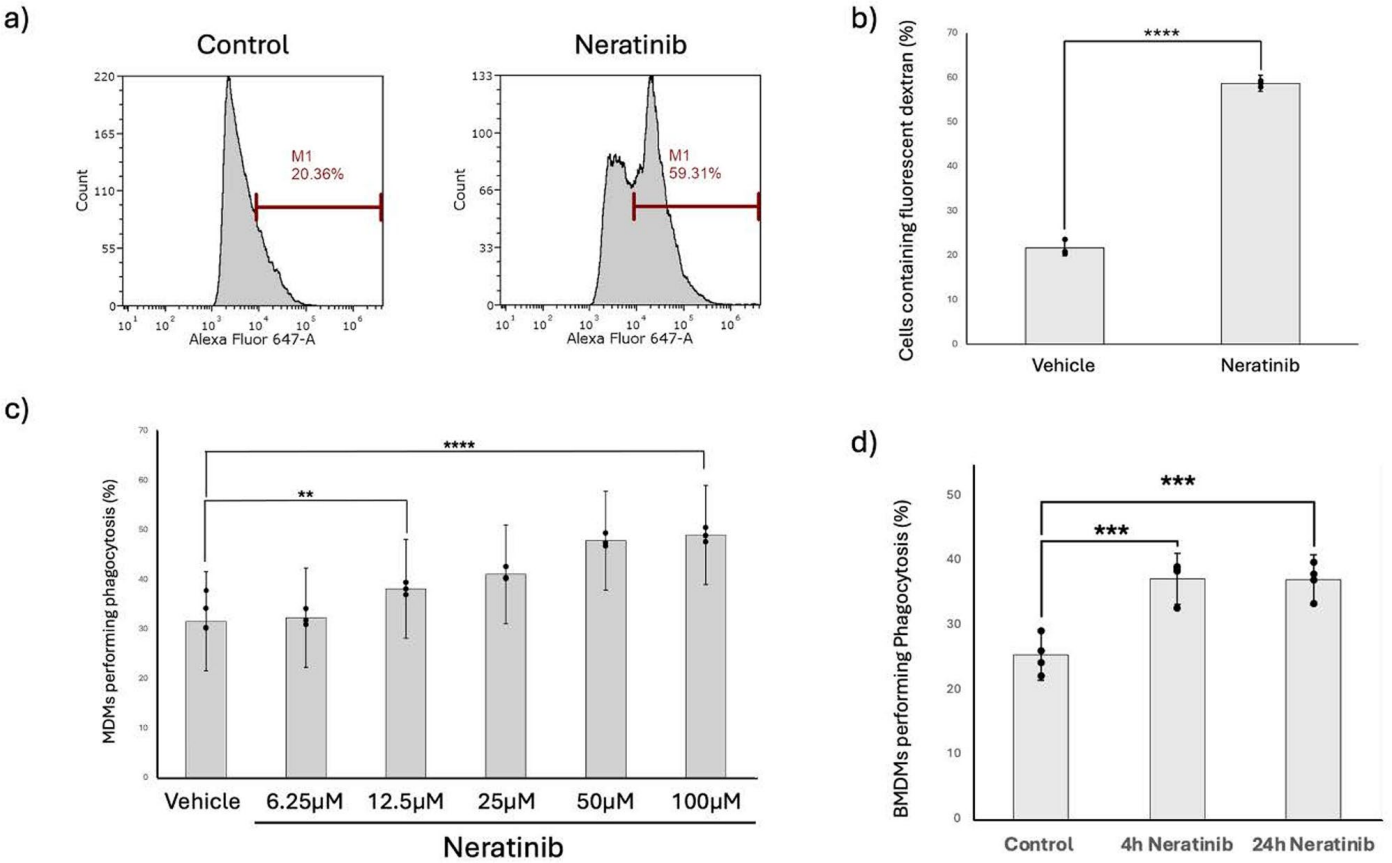
Neratinib significantly increases macrophage macropinocytosis and phagocytosis. MDMs were incubated with DMSO 0.1% (v/v) (vehicle control) or neratinib [20 µM] for 20 h at 37 °C/5% CO_2_ after which fluorescent dextran [100 ng/ml] was added for 1 h and cells analysed by a Cytek Aurora 3 laser flow cytometer (**a**-**b**). (**a**) Representative histograms indicating MDMs containing fluorescent dextran (identified within the gate ‘M1’). (**b**) Graph shows percentage of cells containing fluorescent dextran (*n* = 3). (**c**) MDMs were incubated with DMSO 0.1% (v/v) (vehicle control) or neratinib at the concentrations indicated for 24 h before which fluorescent beads were then added for a further 60 min. (**d**) mouse BMDMs were incubated with neratinib [20 µM] and fluorescent beads were then added for a further 60 min. Phagocytosis was quantified by fluorescent microscopy, counting all cells present and quantifying the percentage of cells which contain fluorescent beads. Graphs indicate mean ± standard deviation. Statistical analysis was performed on 3 (**b**-**c**) or 4 (**d**) independent experiments using an unpaired Students’ t-test (**b**) or one-way ANOVA with Dunnett’s multiple comparisons (**c**-**d**), p values are as indicated. * *p* < 0.05, ** *p* < 0.01, *** *p* < 0.001, **** *p* < 0.0001

### Neratinib induces caspase-dependent human neutrophil apoptosis and efferocytosis by macrophages

We have previously shown that ErbB-targeting TKIs increase human neutrophil apoptosis [13, 14]. Here we show that neratinib significantly increases human neutrophil apoptosis (Fig. 2a-b) which occurs in a caspase-dependent manner as demonstrated by the pan-caspase inhibitor, quinolyl-valyl-O-methylaspartyl-[2,6-difluorophenoxy]-methyl ketone (Q-VD-OPh) (Fig. 2c-d). This suggests neratinib-driven apoptosis will occur in a manner that limits damage to the surrounding tissue, and that cells will be marked for efferocytic clearance by tissue macrophages. Our in vivo data (Fig. 1) and previous work [13, 14] show targeting ErbBs increases efferocytosis in vivo. It is not known however whether this occurs due to an increase in apoptotic neutrophils and therefore there is more available cargo ready to be efferocytosed. To investigate this we studied the direct effect of neratinib on human MDMs in vitro, in experiments where the same number of apoptotic neutrophils were added to each treatment group. Neratinib significantly increased the uptake of annexin-V positive apoptotic human neutrophils by MDMs as determined by fluorescent microscopy (Fig. 3a-c). This was also confirmed by flow cytometry (Fig. 3d). The concentration range used in Fig. 3c was chosen to reflect previous work from our group which observed a significant elevation in neutrophil apoptosis was seen at 25 µM neratinib [14]. The range was extended in this study by including two, two-fold concentrations above and below 25 µM, as the mid-point, to study the effects of macrophage efferocytosis. Interestingly a concentration-dependent effect on efferocytosis was not observed at this timepoint, which may suggest the effects on efferocytosis occur early on and reach a saturation point during prolonged treatment. To understand whether there is an effect of neratinib on other forms of macrophage uptake we also studied macropinocytosis and phagocytosis. MDMs treated with neratinib exhibited increased macropinocytosis of fluorescently labelled dextran (Fig. 4a-b) and increased phagocytosis of fluorescent beads (Fig. 4c). Mouse BMDMs treated with neratinib were also more effective at phagocytosis of fluorescent beads (Fig. 4d). The early (2–6 h) and late (24 h) timepoints were selected to allow us to understand whether neratinib is likely to have a rapid effect on inflammation resolution therapeutically, and also help to point towards the possible mechanisms by which this may occur.

### Neratinib increases expression of MerTK on the cell surface of MDMs

**Fig. 5:**
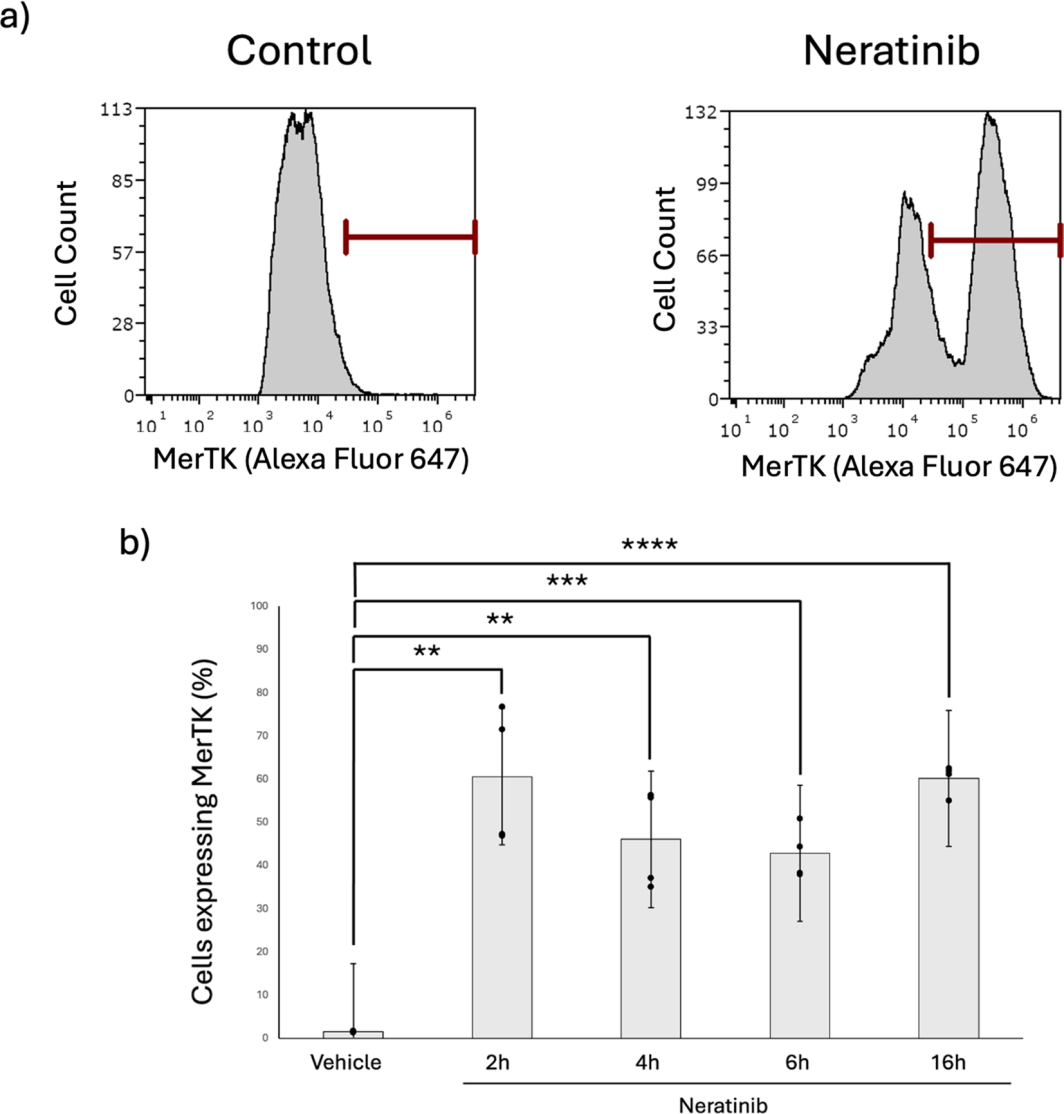
Neratinib upregulates cell surface expression of MerTK on human MDMs. MDMs were incubated with DMSO 0.1% (v/v) (vehicle control) or neratinib [20 µM] at 37 °C/5% CO_2_ for the times indicated. Cells were then stained using an anti-MerTK antibody and analysed with a Cytek Aurora 3 laser flow cytometer. (**a**) Representative flow cytometry histograms show populations of cells staining positively for MerTK within the red marked region. (**b**) Graph showing percentage of MDMs expressing MerTK on the cell surface *n* = 3. Bars indicate mean ± standard deviation. Statistical analysis was performed by using a one-way ANOVA with Dunnett’s multiple comparisons, p values are as indicated. ** *p* < 0.01, *** *p* < 0.001, ****p < 0.0001

**Fig. 6:**
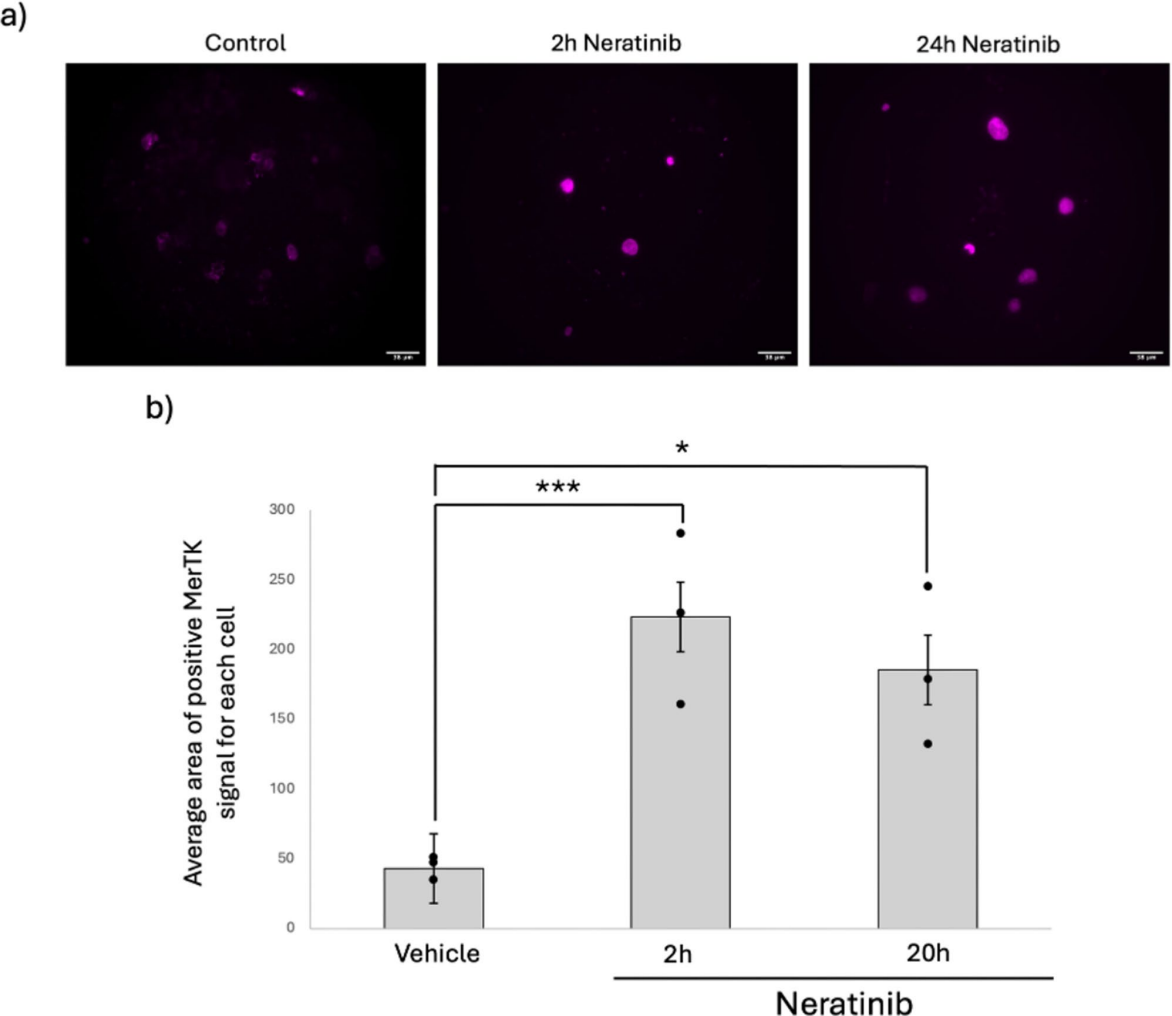
Fluorescent imaging of live MDMs following neratinib treatment to observe MerTK. MDMs were with DMSO 0.1% (v/v) (vehicle control) or neratinib [20 µM] for either 2–20 h at 37 °C/5% CO_2_. MDMs were fluorescently stained using an Alexa Fluor 647 anti-MerTK antibody and imaged using a Nikon Widefield fluorescent microscope. (**a**) Representative images for each treatment group illustrating cells positive for MerTK. (**b**) Graph indicating average area of fluorescence for each cell as an indication of MerTK expression. Statistical analysis was performed by using a one-way ANOVA with Dunnett’s multiple comparisons, P values are as indicated (*n* = 3). * *p* < 0.05, *** *p* < 0.001

**Fig. 7:**
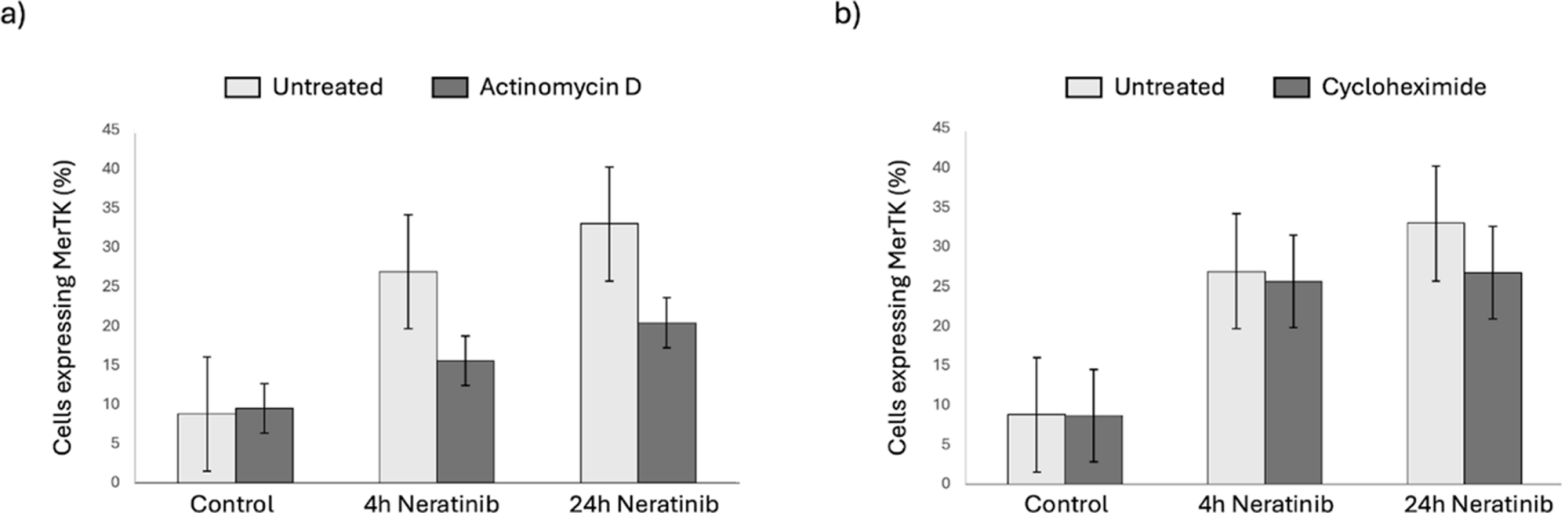
Treating MDMs with actinomycin D or cycloheximide has no significant effect on MerTK expression following neratinib treatment. MDMs were treated with vehicle control (untreated), actinomycin D [5 µg/ml] (**a**) or cycloheximide [100 µg/ml] (**b**) for 1 h, following further treatment for 4–24 h with vehicle control or neratinib [20 µM] at 37 °C/5% CO_2_ (*n* = 3). MDMs were fluorescently stained using a MerTK antibody and analysed by a Cytek Aurora 3 laser flow cytometer. Bars indicate mean, error bars indicate standard deviation. Statistical analysis was performed by using a one-way ANOVA with Dunnett’s multiple comparisons (all non-significant)

We hypothesised that increased efferocytosis was driven by increased surface expression efferocytosis receptors. MerTK is a transmembrane receptor and member of the TAM family and via Gas6 recognises phosphatidylserine on the surface of apoptotic cells to enable apoptotic cell clearance [21]. Flow cytometry showed that neratinib increases the percentage of MDMs expressing cell surface MerTK (Fig. 5a), from timepoints as early as 2 h post neratinib treatment (Fig. 5b). To validate this observation in live cells, fluorescent imaging of live MDMs was performed to quantify the amount of MerTK-derived fluorescent signal for each cell (Fig. 6a). The mean area of fluorescence of the was significantly elevated following neratinib treatment (Fig. 6b), indicating more MerTK expression per cell, which is consistent with increases in MFI observed in flow cytometry data (data not shown). To determine if the increase in MerTK expression was as a result of *de novo* MerTK expression we used inhibitors of transcription (actinomycin D) and translation (cycloheximide). Neither actinomycin D (Fig. 7a) nor cycloheximide (Fig. 7b) significantly modified neratinib-induced MerTK expression. This finding suggests that neither transcription nor translation is responsible for the elevated MerTK levels observed on MDMs following neratinib treatment.

### Neratinib-induced efferocytosis is dependent on MerTK

**Fig. 8:**
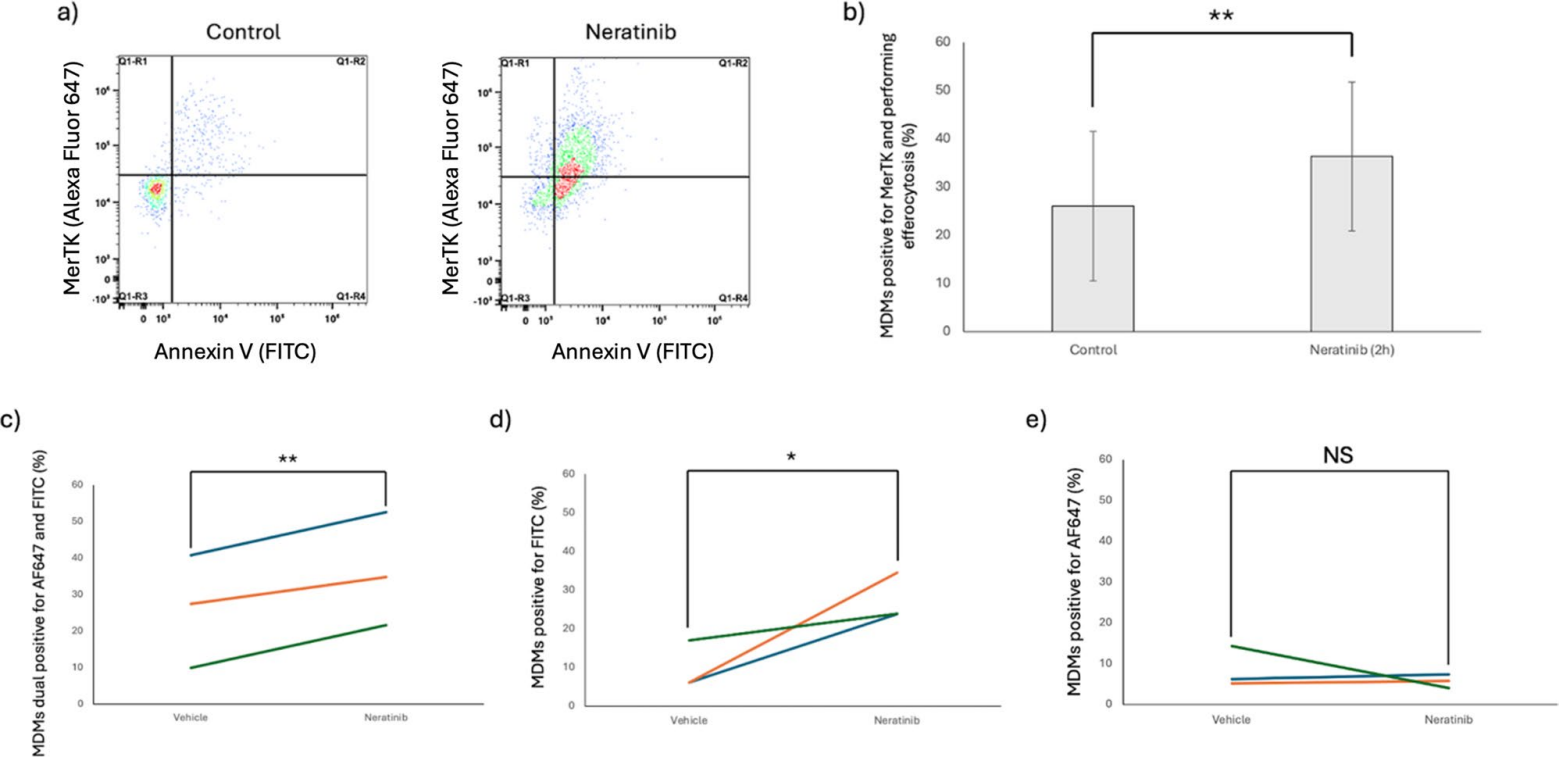
Neratinib-induced MerTK expression correlates with elevated efferocytosis in MDMs. MDMs were incubated with DMSO 0.1% (v/v) (vehicle control) or neratinib [20 µM] for 2 h at 37 °C/5% CO_2_ following which they were incubated with annexin-V (FITC) stained apoptotic neutrophils for 1 h. MDMs were then stained with an anti-MerTK antibody (Alexa Fluor 647) before being analysed by a Cytek Aurora 3 laser flow cytometer (*n* = 3). (**a**) Representative flow cytometry dot plots showing staining profiles for single (Q1-R1, Q1-R4) and dual stained (Q1-R2) cells. (**b**) Graph showing mean ± standard deviation cells expressing MerTK and performing efferocytosis (dual positive, Q1-R2). Graphs show change in individual experiments for cells that are dual positive (**c**), single positive for efferocytosis (**d**) and single positive for MerTK (**e**). Statistical analysis was performed by using a Students’ t test, p values are as indicated *p < 0.05, **p < 0.01

**Fig. 9:**
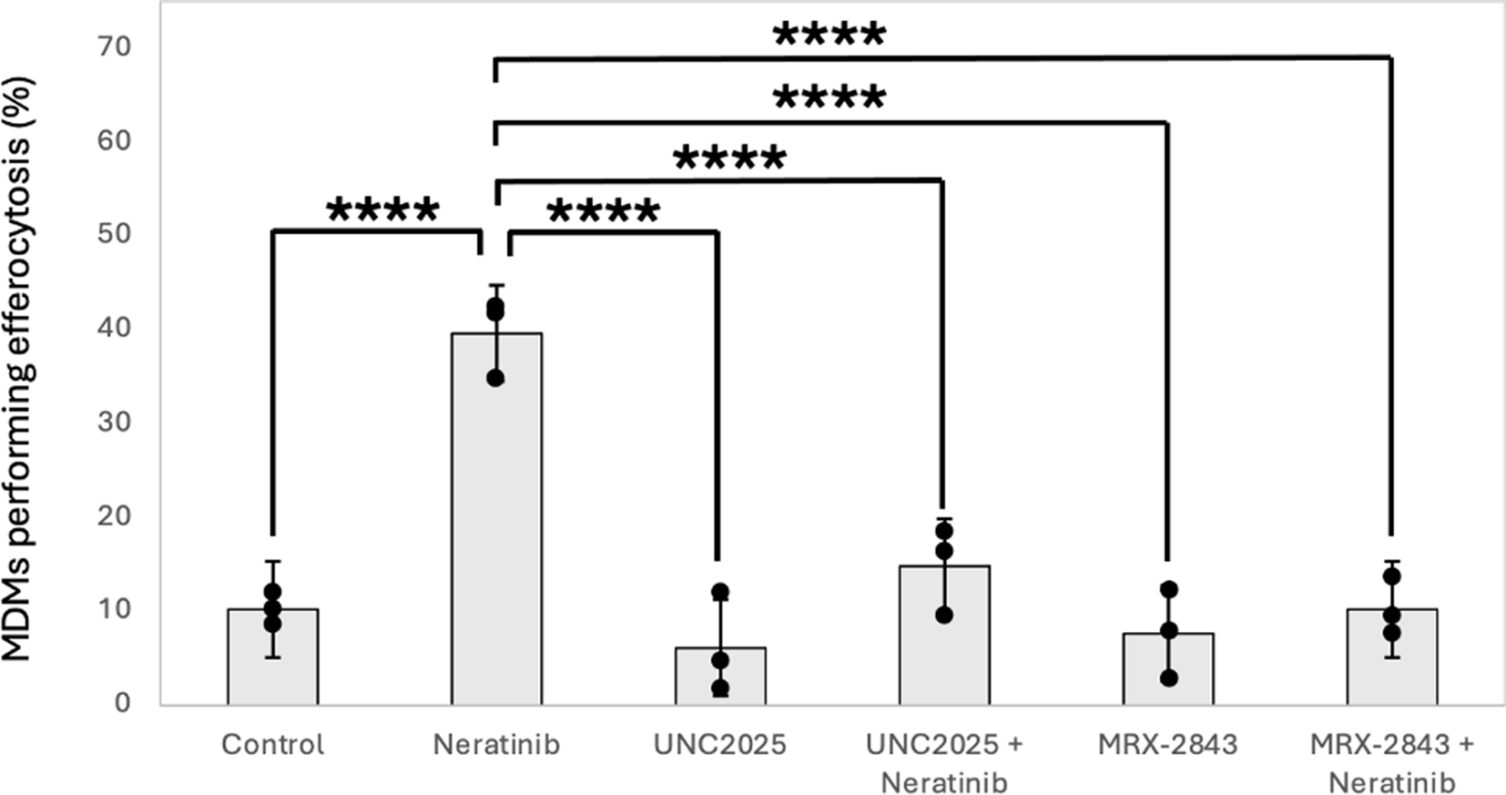
Neratinib-induced efferocytosis is dependent on MerTK. MDMs were incubated with DMSO 0.1% (v/v) (vehicle control), or neratinib [20 µM], as indicated for 20 h at 37 °C/5% CO_2,_ following a 2 h pre-treatment with the MerTK inhibitors UNC2025 [1 µM], or MRX-2843 [150 nM]. MDMs were subsequently incubated with annexin-V (PE, red) stained apoptotic neutrophils for 1 h. MDMs were stained with FITC-Phalloidin and efferocytosis measured by fluorescent microscopy. Graph shows mean percentage of MDMs performing efferocytosis, error bars are standard deviation (*n* = 3). Statistical analysis was performed by using a one-way ANOVA with Dunnett’s multiple comparisons, ****: *p* < 0.0001

To understand the relationship between increased MerTK expression and increased efferocytosis, both were measured by flow cytometry in the same population of cells in order to identify single positive cells (cells positive for either MerTK or performing efferocytosis) and dual positive cells (cells positive for both MerTK and performing efferocytosis). There was an increase in dual positive cells following neratinib treatment (Fig. 8b-c) and this was the majority cell population (mean % for control; 26.06% and neratinib; 36.32%). There is also a significant increase in single positive cells that performed efferocytosis that were negative for MerTK expression (mean % for control; 9.74% and neratinib; 27.38%) (Fig. 8b). There was no change in the percentage of single positive cells expressing MerTK that were not performing efferocytosis (mean % for control; 8.56% and neratinib; 5.68%) (Fig. 8e). Overall, this suggested that an increase in MerTK expression translates to an increase in efferocytosis, but that efferocytosis can take place either in the absence of MerTK, or that MerTK expression has somehow been lost from the cell. MerTK inhibitors were used to better understand whether there was a causal effect of MerTK in increasing efferocytosis. UNC2025 and MRX-2843 are closely related small molecule MerTK inhibitors which block the ATP-binding site of MerTK to prevent downstream signalling [22, 23]. Inhibiting MerTK using these compounds significantly reduced the efferocytosis rates of MDMs following neratinib treatment down to control levels (Fig. 9), suggesting that elevated MerTK expression is responsible for the increase in efferocytosis.

## Discussion

**Fig. 10:**
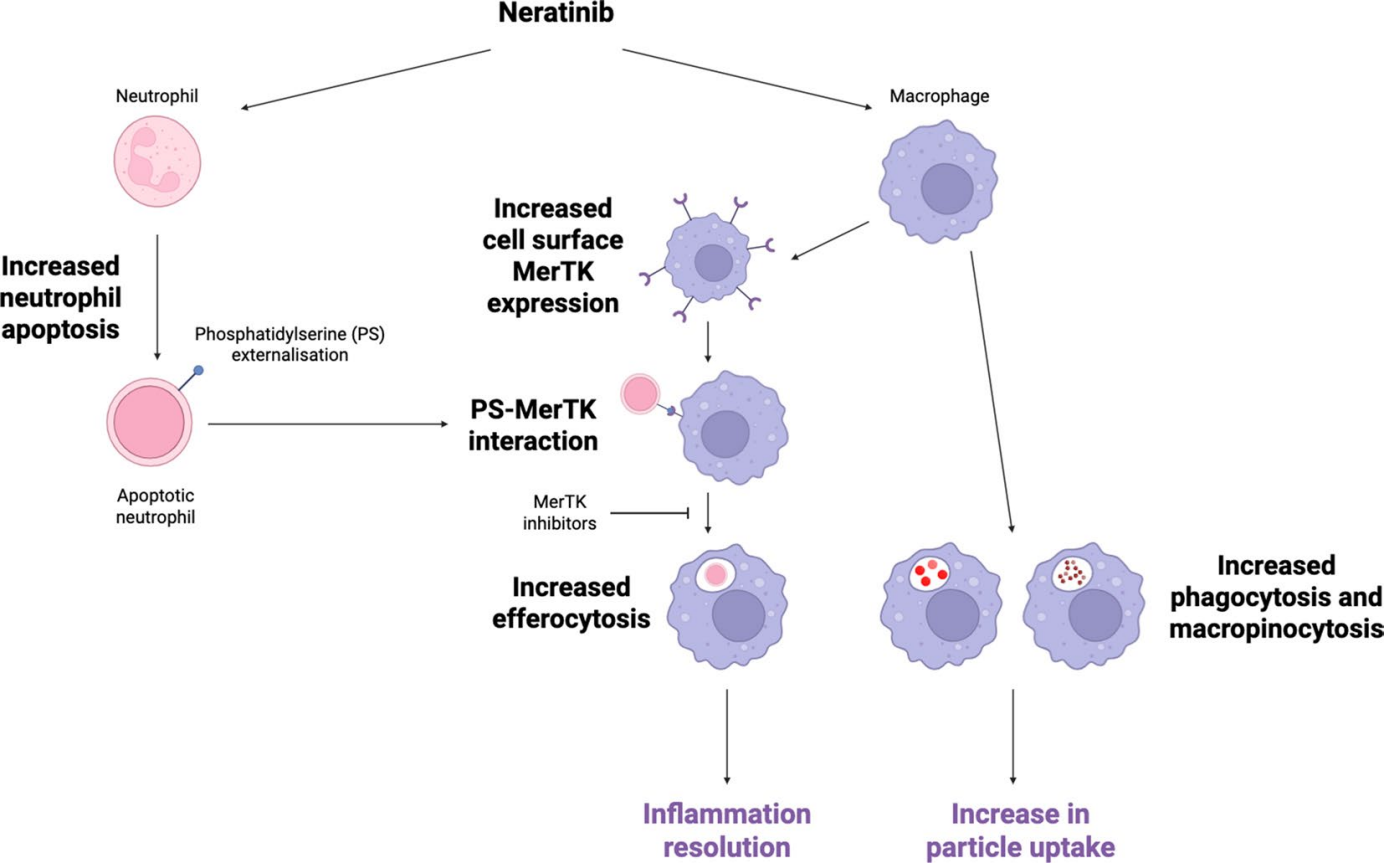
The parallel anti-inflammatory effects of neratinib on neutrophils and macrophages. Neratinib increases neutrophil apoptosis leading to externalisation of phosphatidylserine (PS). In parallel, neratinib acts on macrophages to increase cell surface expression of MerTK, which can then interact with PS on apoptotic neutrophils resulting in increased efferocytosis. Neratinib also upregulates uptake of fluorescent beads and fluorescent dextran by macrophages. Together these events have the potential to promote inflammation resolution in disease. Made with Biorender.com

The perpetuation of the inflammatory response and the elevated presence of neutrophils in the tissues is an important factor in COPD and atherosclerosis pathology [24–29]. Efferocytosis is a critical step in the efficient clearance of neutrophils following apoptosis in order for effective inflammation resolution to take place. Previously we identified novel anti-inflammatory effects of ErbB-targeting compounds in murine and zebrafish models of inflammation and in inflammatory cells in vitro [13, 14]. Here we show for the first time that neratinib, an ErbB-targeting drug used to treat breast cancer, increases efferocytosis by MDMs and airway macrophages in multimorbid mice. The increase in MDM efferocytosis in vitro indicates that neratinib is acting directly on macrophages, rather than simply increasing the number of apoptotic neutrophils available to efferocytose. This, alongside the parallel effects on neutrophils to drive apoptosis, gives neratinib the potential to be a powerful therapeutic strategy, particularly in conditions where there is both a persistence of neutrophils and a defect in efferocytosis, such as COPD [6, 7]. A further potential benefit of neratinib is its ability to promote cellular uptake more widely via macropinocytosis (used to monitor the extracellular environment) and phagocytosis (to clear pathogens and other particulates from the lung). Macrophages isolated from people with COPD show reduced phagocytic responses to bacteria, and increasing these cellular processes may have a positive impact on response to infection, which is a common cause of exacerbation in COPD [30, 31]. The main findings of the paper are summarised in Fig. 10.

Since our overall aim is to identify new therapeutic strategies, we have focussed on using a drug which could be reprofiled for use in inflammatory disease. We acknowledge the limitations of an entirely pharmacological approach, which includes potential off target effects via non-ErbB pathways. Although it is not possible to genetically modify human neutrophils, we have previously recapitulated effects of ErbB-TKIs in *egfra* and *erbb2* CRISPR-modified zebrafish in previous studies [13]. In further support of our work, a recent study elegantly shows that myeloid-specific deletion of EGFR promotes a pro-resolving, anti-inflammatory phenotype and increased efferocytosis by macrophages in a murine model of ischemic acute kidney injury [32].

We explored the possible mechanisms by which neratinib upregulates efferocytosis and found a significant increase in MerTK expression. MerTK is the predominant efferocytic receptor in many tissues and is key to facilitating macrophage efferocytosis of neutrophils including in the lung [33–35]. Via Gas6, MerTK binds to the cell surface ‘eat-me’ receptor, phosphatidylserine on apoptotic cells, which initiates cytoskeletal rearrangements to allow the formation of a phagocytic cup around the apoptotic cell and efferosome maturation [21]. The importance of MerTK-mediated efferocytosis by lung macrophages in the prevention of inflammation has been demonstrated by others [35]. However, here we show an increase in the proportion of MDMs in a population that express MerTK, as well as an increase in the amount of MerTK per cell. Since this increase was detected at timepoints as early as 2 h, we hypothesised it was independent of transcription and translation, and experiments using actinomycin D and cycloheximide confirmed this was indeed the case. MerTK expression and function is tightly regulated to avoid over-activation, for example via metalloprotease (MMP) cleavage [36, 37]. It is possible that neratinib prevents MMP-mediated cleavage of MerTK, or that it is being mobilised from other cell compartments, and future studies are required to study this. Using dual-stain flow cytometry we were able to show that neratinib significantly increased the proportion of MDMs that were both positive for MerTK and efferocytosed apoptotic neutrophils, as well as increasing the proportion of MDMs that were negative for MerTK and efferocytosed apoptotic neutrophils. This suggests that either MerTK has been lost on some cells, or that it is not an absolute requirement for MDMs to perform efferocytosis and other receptors are possibly at play. There was no change in the proportion of MDMs that expressed MerTK but had not efferocytosed apoptotic cells, which suggests that MerTK is likely to play a key role in this process. To better understand this, we used the closely related MerTK inhibitors MRX2843 and UNC2025 in MDM efferocytosis assays and show that inhibiting MerTK reduces neratinib-induced efferocytosis down to baseline levels. This indicates that neratinib-induced efferocytosis is dependent on MerTK activity.

Macrophage phagocytosis is impaired in many chronic inflammatory diseases including COPD, resulting in reduced clearance of pathogens from the lungs and an increase in exacerbation rate and severity [38]. Our phagocytosis studies suggest neratinib may have potential benefit in the context of infection, although further studies using infection models would be required to better understand this. Since macropinocytosis and uptake of beads involve a number of receptor pathways it is possible that targeting ErbBs regulates global mechanisms relating to phagocytosis. Our previous phosphoproteomic analysis in neutrophils identified an enrichment in the Rho GTPase cycle, a known controller of efferocytosis and phagocytosis [14], and exploring the Rho GTPase-regulated F-actin recruitment and re-arrangement is worthy of future study [39].

The treatment of COPD focuses on the alleviation of symptoms, mainly through combinations of inhaled bronchodilators and corticosteroids, as well as strategies to prevent exacerbations, for example via phosphodiesterase inhibition and antibiotics [40]. There is a great need to shift towards personalised, targeted therapy and this is the focus of ongoing clinical studies [41]. COPD is a common component of multimorbidity, and we demonstrate effects of neratinib in a combined murine model of atherosclerosis and acute lung disease, suggesting this drug may have benefits even where there is a complex inflammatory background. Furthermore, we show effects when neratinib is given at the same time as the inflammatory stimulus (and 24 h post-inflammatory stimulus) suggesting there could be therapeutic benefit once inflammation is established, for example during a COPD exacerbation.

## Data Availability

The data that support the findings of this study are not openly available but are available from the corresponding author upon reasonable request.
